# Supplementary material for: Factors of parental COVID-19 vaccine hesitancy: A cross sectional study in Japan
Source: PLoS One. 2021 Dec 17;16(12):e0261121. doi: 10.1371/journal.pone.0261121 (PMC8683027; doi:10.1371/journal.pone.0261121)
Supplement: S1 File — (DOCX) [file pone.0261121.s001.docx]

**[web survey (2021-05)].**

| **Survey on child's health and life during the COVID-19 outbreak** |
| --- |

| **First, let us ask you about your gender and age.** |
| --- |

**[For all]**

SC1. Please tell us your gender (only one).

| **1** Female  **2** Male |
| --- |

[Page Break]

SC2. Please tell us your age (only one).

| **1** Under 20 years old  **2** 20–24 years old  **3** 25–29 years old  **4** 30–34 years old  **5** 35–39 years old  **6** 40–44 years old  **7** Over 45 years old |
| --- |

[Page Break]

SC3. Do you have your own children who currently live with you? If so, please indicate the gender and age of the child(ren) (all that apply).

| **1** No children live together ( → Survey completed)  **2** Boys under 2 years old  **3** 3-year-old boy (younger class in kindergarten/nursery in April)  **4** 4-year-old boy (year-round class for kindergarten and nursery school in April)  **5** 5 -year-old boy (senior class of kindergarten and nursery school in April)  **6** 6-year-old boy (1st grade in elementary school in April)  **7** 7-year-old boy (2nd grade in elementary school in April)  **8** 8-year-old boy (3rd grade in elementary school in April)  **9** 9-year-old boy (4th grade in elementary school in April)  **10** 10-year-old boy (5th grade in elementary school in April)  **11** 11-year-old boy (6th grade in elementary school in April)  **12** 12-year-old boy (1st grade in junior high school in April)  **13** 13-year-old boy (2nd grade in junior high school in April)  **14** 14-year-old boy (3rd grade in junior high school in April)  **15** ≥15-year-old boy (high school and above in April)  **16** 2-year-old girl  **17** 3-year-old girl (younger class in kindergarten/nursery in April)  **18** 4-year-old girl (year-round class for kindergarten and nursery school in April)  **19** 5-year-old girl (senior class of kindergarten and nursery school in April)  **20** 6-year-old woman of child (1st grade in elementary school in April  **21** 7-year-old woman of child (2nd grade in elementary school in April)  **22** 8-year-old girl (3rd grade in elementary school in April)  **23** 9-year-old girl (4th grade in elementary school in April)  **24** 10-year-old girl (5th grade in elementary school in April)  **25** 11-year-old girl (6th grade in elementary school in April)  **26** 12-year-old girl (1st grade in junior high school in April)  **27** 13-year-old girl (2nd grade in junior high school in April)  **28** 14-year-old girl (3rd year in junior high school in April)  **29** ≥15-year-old girl (high school students and above in April) |
| --- |

Cohabitation check: Option 1 and 2 to 29 simultaneous answers → Alert

[Page Break]

[For those who chose either 3–14 or 17–28 in SC3 (there is at least one boy/girl aged 3-14 years)].

SC4. Please read the following and answer the following sentences carefully if you agree to complete this questionnaire.

**[Request for cooperation with survey]**

This survey is conducted by Nippon Research Center and commissioned by the Center for Birth Cohort Studies, University of Yamanashi.

The COVID-19 countermeasures have placed great strain on the mental health of children due to the stress of staying at home and loneliness of not being able to meet others. There is an urgent need to prevent child psychological issues while taking countermeasures against COVID-19.

Therefore, the Center for Birth Cohort Studies, University of Yamanashi, is conducting this survey to grasp the current situation of children and parents during the COVID-19 outbreak, and to use the results to advise future countermeasures. Therefore, the Center for Birth Cohort Studies, University of Yamanashi, conducts this survey to grasp the current situation of children and parents, and to use the results to advise future countermeasures. The results of this survey will be analyzed and reported in academic conferences and journals.

　We appreciate your cooperation with this survey.

In this survey, we will ask you about the health condition of you and your child, but your answers will be anonymized and used only for research at the Center for Birth Cohort Studies, University of Yamanashi. We will never use your name or answer for other purposes.

Do you agree to participate in this survey? (Only one)

| **1** Yes, I do (→ Go to SC1)  **2** No, I do not ( → Survey completed) |
| --- |

[Page Break]

Only those who chose "1" in SC 4 move to the survey section (Q1)

| **Please answer each of the following questions.** |
| --- |

Q1. Please indicate in which prefecture you currently live.

| **1** Hokkaido  **2** Aomori  **3** Iwate  **4** Miyagi  **5** Akita  **6** Yamagata  **7** Fukushima  **8** Ibaraki  **9** Tochigi  **10** Gunma  **11** Saitama  **12** Chiba  **13** Tokyo  **14** Kanagawa  **15** Niigata  **16** Toyama  **17** Ishikawa  **18** Fukui  **19** Yamanashi  **20** Nagano  **21** Gifu  **22** Shizuoka  **23** Aichi | **24** Mie  **25** Shiga  **26** Kyoto  **27** Osaka  **28** Hyogo  **29** Nara  **30** Wakayama  **31** Tottori  **32** Shimane  **33** Okayama  **34** Hiroshima  **35** Yamaguchi  **36** Tokushima  **37** Kagawa  **38** Ehime  **39** Kochi  **40** Fukuoka  **41** Saga  **42** Nagasaki  **43** Kumamoto  **44** Oita  **45** Miyazaki  **46** Kagoshima  **47** Okinawa |
| --- | --- |

[Page Break]

Q2. What is the size of the city you currently live in?

| **1** More than 500,000  **2** 300,000 to less than 500,000 people  **3** 100,000 to less than 300,000  **4** 50,000 people to less than 100,000 people  **5** 30,000 to less than 50,000 people  **6** 10,000 to less than 30,000  **7** Less than 10,000 |
| --- |

[Page Break]

Q3. Was a state of emergency declared or was an area for priority measures to prevent the spread of the disease designated in the area where you live? (Please select only one)

| 1. Specified 2. Not specified. |
| --- |

[page break]

Q4. Please tell us about all the people who currently live with you and share your livelihood (household members) other than your children. (Choose as many as you like)

| 1. Partner (including spouse) 2. Your father. 3. Your mother. 4. Partner's father 5. Partner's mother 6. Other ( ) 7. No family members other than children living together and making a living together |
| --- |

Co-residence check: Answer options 1 to 6 and 7 at the same time → Alert

[Page Break]

Q5. How many children do you have? Please tell us how many children you have, regardless of whether they live with you or not. (Choose only one).

| 1. One 2. Two 3. Three 4. Four 5. Five 6. Six or more |
| --- |

[Page Break]

| **The following questions are about yourself and your ●-year-old ● (boy/girl) child living together.** |
| --- |

　　* " ● " indicates the answer to SC3 (if there are multiple answers, it is randomly chosen.)

The same applies to the following.

Q6-1. Please tell us about your child**'**s attendance at preschool or school during the past month**. Has your child been attending** preschool or school on weekdays during the past month? (Only one)

| 1. Attends school almost every day 2. Has missed 4 or more days in the month. 3. Not attending preschool or school |
| --- |

[Page Break]

**(For those who answered "1 Attends preschool or school almost every day" or "2 Has missed 4 or more days in the month" in Q6-1)**

Q6-2. Please tell us about your child's condition at preschool or school**.**

　　　　　(only one)

| 1. No change compared to before the COVID-19 outbreak started in February 2020. 2. They seem to be enjoying going more compared to before the COVID-19 outbreak started in February 2020. 3. Not having as much fun going as before the COVID-19 outbreak started in February 2020. 4. None of the above. |
| --- |

[page break]

**(For those who answered "Has missed 4 or more days in the month" or "Not attending preschool or school" in Q6-1)**

Q6-3. Who decided that your child **"a child ●years old●**” will not attend preschool or school?

　　　　　Please select the one that best applies: (Choose only one).

| 1. Your child themself 2. You (the respondent of this survey) 3. Partner (including spouse) 4. Attending physician 5. Other ( ) |
| --- |

[Page Break]

**(For those who answered "Has missed 4 or more days in the month" or "Not attending preschool or school" in Q6-1)**

Q6-4. Please tell us why your child **"a child of ●years old●” did** not attend preschool or school.

　　　　　Please choose one of the following applies to you: (Choose as many as you like? )

| 1. The school is closed 2. Due to the child's own underlying medical condition 3. Due to the child's own physical condition 4. Due to the child's own mental health problems 5. Other ( ) |
| --- |

[page break]

Q7. Is your child able to wash their hands and perform appropriate coughing etiquette?

| **1** Perfectly  **2** Almost perfectly  **3** OK  **4** Not very well  **5** No |
| --- |

[Page Break]

Q8. How often does your child play outside?

| **1** Almost every day  **2** Three to five days per week  **3** One or two days per week  **4** Hardly goes outside |
| --- |

[Page Break]

Q9-1. How is your child's use of LINE, games, YouTube, etc., during the past month compared with that before the COVID-19 outbreak started in February 2020? (Choose only one).

| 1. More than three times 2. Approximately two times more 3. Approximately the same amount of usage 4. Less time spent using the system 5. No use |
| --- |

[page break]

**[For those who answered "1-4" in Q9-1].**

Q9-2. Please tell us about amount of time your child spends watching LINE, games, YouTube, etc. per day in the past month (answer with numbers).

　　hours per day

　　 　　(Limit the answer from 1 to 24)

[New Page]

The following questions are related to the mental health of your child, which was introduced by the Ministry of Health, Labor, and Welfare. Please answer for your **"● year-old ●"**

Various signs appear when your worries and stress grow. It is important to be aware of signs that are different from usual, such as "this has never happened before" and "it looks different than usual". If you see the following signs, talk to your child: If you continue to see the symptoms, it might be worth consulting a specialist.

Please choose all that apply.

Sleep: Good sleep and good sleep are important for your mental health.

| Even if he/she goes to bed, it seems that he/she can't sleep easily. | one |
| --- | --- |
| He/She stays up until late. | two |
| He/She finds it hard to get up in the morning. | three |
| The rhythm of sleep is broken. | four |
| He/She says "I can't sleep". | five |
| He/She sleeps too much. | six |

Appetite: Stress and mental illness can affect appetite.

| He/She has no appetite and eats less. | seven |
| --- | --- |
| He/She eats too much. | eight |
| He/She wants carbohydrates, such as bread, rice, and sweets. | nine |
| He/She has lost or gained weight dramatically. | ten |
| He/She is very concerned about his/her weight. | 11 |

Physical condition: Mental illnesses often first appears with changes in physical condition.

| He/She feels uncomfortable. | 12 |
| --- | --- |
| He/She looks tired. | 13 |
| He/She is not feeling well. | 14 |
| He/She looks sick. | 15 |
| He/She complains of abdominal pain, headache, dizziness, and nausea. | 16 |

Behavior: Behavioral signs are easier for others to notice than the person in question.

| He/She doesn't want to go to school. | 17 |
| --- | --- |
| He/She shuts him/herself in at home. | 18 |
| He/She stopped playing with his/her friends. | 19 |
| He/She doesn't care about his/her appearance. | 20 |
| He/She is often silent. | 21 |
| He/She has stopped greeting others. | 22 |
| He/She repeats the same actions. | 23 |
| He/She can't control his/her feelings and uses violence. | 24 |
| He/She has been absent-minded for a long time without doing anything. | 25 |
| His/her facial expression does not change, and his/her emotional reactions have decreased. | 26 |
| His/Her speech has become incoherent and incomprehensible. | 27 |
| He/She has started to talk to him/herself. | 28 |

| None of the above | 29 |
| --- | --- |

The above signs do not necessarily mean that your child has a mental illness. However, if you can see such a sign, or if the sign lasts for a long time, it may be a warning from your child.

Earlier intervention will lead to faster recovery from mental illness. Therefore, if you find a child exhibiting "unusual" signs, consult a specialist as soon as possible.

[Page Break]

| **Next, let's talk about you and your family.** |
| --- |

Q11. Please tell us about your and your partner’s (wife, husband, etc.) working status.

Q11-1. what is your job? (Only one)

| **1** Office worker  **2** Civil servant  **3** Self-employed/freelance  **4** Agriculture, forestry, and fisheries  **5** Part-time job  **6** Full-time housework (home)  **7** Student  **8** Unemployed  **9** Other ( ) |
| --- |

[Page Break]

**For those who answered "1-5" in Q11-1.**

Q11-2. Please tell us about your work situation (telework implementation status) before the COVID-19 outbreak in February 2020. (Choose only one).

| 1. I teleworked or otherwise worked from home almost every day. 2. I teleworked or otherwise worked from home about half the week. 3. I commuted to and from work almost every day (including self-employment) 4. I wasn't working. |
| --- |

**For those who answered "1-5" in Q11-1.**

Q11-3. Please tell us about your current work situation (telework implementation status). (Choose only one).

| 1. I telework or otherwise work from home almost every day. 2. I telework or otherwise work from home about half the week. 3. I commute to and from work almost every day (including self-employment) |
| --- |

[page break]

Q11-4. What is your partner's job? (Only one)

| **1** Office worker  **2** Civil servant  **3** Self-employed/freelance  **4** Agriculture, forestry, and fisheries  **5** Part part-time job  **6** Full-time housework (home)  **7** Students  **8** Unemployed  **9** Others ( )  **10** No partner/spouse |
| --- |

[Page Break]

**For those who answered "1-5" in Q11-4.**

Q11-5. Please tell us about your partner's (including spouse's) work situation (teleworking status) before the COVID-19 outbreak in February 2020. (Choose only one).

| 1. He/She teleworked or otherwise worked from home almost every day. 2. He/She teleworked or otherwise worked from home about half the week. 3. He/She commuted to and from work almost every day (including self-employment) 4. He/She wasn't working. |
| --- |

**For those who answered "1-5" in Q11-4.**

Q11-6 Please tell us about your partner's (including spouse's) current work situation (telework implementation status). (Choose only one).

| 1. He/She teleworks or otherwise works from home almost every day. 2. He/She teleworks or otherwise works from home about half the week. 3. He/She commutes to and from work almost every day (including self-employment) |
| --- |

[page break]

Q12. Please tell us about your household income in the previous year. (Please choose only one)

| 1. Less than 2 million yen 2. More than 2 million yen but less than 4 million yen 3. More than 4 million yen but less than 6 million yen 4. Over 6 million yen 5. I don't know. |
| --- |

[page break]

Q13-1. Please tell us about the impact of the COVID-19 outbreak on your employment.

(HOW MANY? )

| 1. I was hired out. 2. I quit my job voluntarily. 3. I found a new job. 4. My income increased. 5. My income decreased. 6. Nothing has changed. |
| --- |

Co-residence check: Answer options 1-5 and 6 at the same time → Alert

[page break]

**For those who answered "1-9" in Q11-4.**

Q13-2. Please tell us about the impact of the COVID-19 outbreak on your partner's (including spouse's) employment. (Choose as many as you like)

| 1. He/She was hired out. 2. He/She quit his/her job voluntarily. 3. He/She found a new job. 4. His/Her income increased. 5. His/Her income decreased. 6. Nothing has changed. |
| --- |

Co-residence check: Answer options 1-5 and 6 at the same time → Alert

[page break]

Q14. What are your concerns? Please choose all that apply.

| **1** My work/housework  **2** Partner’s (wife, husband, etc.) work/housework  **3** Have a child attend a nursery/school  **4** Relieve your stress  **5** Relieve your partner's (wife, husband, etc.) stress  **6** Education of your child  **7** Your child’s lack of exercise  **8** Psychological stress of your child  **9** Home meals  **10** Household income and economic situation  **11** Difficulty in getting masks  **12** Increased troubles at home  **13** Others ( )  **14** I have no problems. |
| --- |

[Page Break]

Q15. Please answer the following questions. This questionnaire evaluates psychological stress and was also conducted in the Comprehensive Survey of Living Conditions. Please tell us about this recent situation. (Choose one for each)

| During the past 30 days, about how often did you feel ... | All of the time | Most of the time | Some of the time | A little of the time | None of the time |
| --- | --- | --- | --- | --- | --- |
| 1. ...nervous? | one | two | three | four | five |
| 1. ...hopeless? | one | two | three | four | five |
| 1. ...restless or fidgety? | one | two | three | four | five |
| 1. ...so depressed that nothing could cheer you up? | one | two | three | four | five |
| e. ...that everything was an effort? | one | two | three | four | five |
| f. ...worthless? | one | two | three | four | five |

[Page Break]

Q16. Which of the following concern you about SARS-CoV-2? Please select all that apply.

| 1. I'm worried that I might be infected with SARS-CoV-2. 2. I'm worried that I won't be able to protect my family from SARS-CoV-2. 3. I am afraid that the healthcare system in my country will not be able to protect my loved ones from SARS-CoV-2. 4. I am afraid that the healthcare system in my country is not able to protect me from SARS-CoV-2. 5. I am concerned that basic preventive measures (hand-washing, masks) and avoiding densely populated areas may not be enough to protect against SARS-CoV-2. 6. I'm concerned about the infectivity of mutant viruses. 7. I'm concerned that mutant viruses may be more likely to cause severe disease. 8. I am concerned that mutant viruses may be more likely to affect children. 9. None of the above. |
| --- |

Co-residence check: Answer options 1-8 and 9 at the same time → Alert

[page break]

Q17-1. How satisfied are you with your current social connections, such as friendships and the community? Please select only one.

| 1. 10 points (very satisfied) 2. 9 points 3. 8 points 4. 7 points 5. 6 points 6. 5 points 7. 4 points 8. 3 points 9. 2 points 10. 1 point (not satisfied at all) |
| --- |

[Page Break]

Q17-2. Which of the following greatly influences your satisfaction or dissatisfaction with your current social ties, such as friendships and the community? Please select all that apply.

| 1. Family and relatives who can be relied on in times of trouble 2. Friends and acquaintances who can be relied on in times of trouble 3. Colleagues and superiors at work who can be relied on in times of trouble 4. Frequency of interaction with friends 5. Opportunities to participate in volunteer activities, neighborhood associations, and other activities 6. Connections on social networking sites (Facebook, Twitter, LINE, etc.) 7. Opportunities to use public facilities and services (e.g., municipal consultation services, health classes, etc.) 8. Other ( ) 9. None of the above |
| --- |

Co-residence check: Answer options 1-8 and 9 at the same time → Alert

[page break]

Q18. Which of the following sources of information on COVID-19 do you trust the most? (Choose only one).

| 1. Information disseminated by government agencies (government, ministries, public health centers, infectious disease laboratories, etc.) 2. Reports by NHK or online news 3. Private reporting or online news 4. Social media (Twitter, Instagram, Facebook, etc.) 5. Information from people close to you 6. Other ( ) 7. None of the above |
| --- |

[page break]

Q19-1. Which of the following applies to you regarding COVID-19 vaccines? (Choose only one).

| 1. I want to be vaccinated. 2. I don't want to be vaccinated. |
| --- |

[page break]

**(For those who answered "2 I don’t want to be vaccinated" in Q19-1)**

Q19-2. If you answered that you don’t want to be vaccinated, please choose one reason that best describes why.

| 1. Because there are doubts about the effectiveness of the vaccine against SARS-CoV-2 infection 2. I'm afraid of adverse reactions 3. I'm not a fan of the vaccine itself 4. Other ( ) |
| --- |

[Page Break]

Q20. If a COVID-19 vaccine became available for children, which applies to you? (Choose only one).

| 1. I want my child to be vaccinated 2. I don't want my child to be vaccinated. |
| --- |

[page break]

Q21. Please write anything that you think about the COVID-19 countermeasures.

|  |
| --- |

[New Page]

This is the end of the survey. We appreciate your cooperation.
