# Supplementary material for: Factors of parental COVID-19 vaccine hesitancy: A cross sectional study in Japan
Source: PLoS One. 2021 Dec 17;16(12):e0261121. doi: 10.1371/journal.pone.0261121 (PMC8683027; doi:10.1371/journal.pone.0261121)
Supplement: Attachment — Submitted filename: Reviewer comments.docx [file pone.0261121.s003.docx]

This subject is absolutely essential for disease prevention and socio-behavioral health.

**Introduction**

Well written

**Methods**

The participant section provides the detailed registration system of the Nippon Research Center. What is the objective of the registration? Which types of communities are registering? Are both parents reporting? Till the study, how many registered? As it is voluntary, registration may be for those who have positive motivation, they registered. How did you justify your representation of sampling? If possible, clarify, otherwise mention this in limitation.

In the data collection section: 1200 is a good sample. How did you select this sample from the register? What is your target population?

**Results**

In line 180

Intention to vaccinate themselves (parent), since adult vaccination started long back, it is good to presented vaccinated versus non-vaccinated (why intention to vaccinate) – present parent practice rather than their attitude. Then you can associate parent vaccination status with intension to vaccinate their child.

Table 3. Reason of having no intention to vaccinate themselves (N = 315) – present the finding using a figure (pi chart).

Combine Table 1 and Table 4 (The variable which described in one table describe it in text)

Table 1. Characteristics of study parents in Japan (year 2021) (N = 1,200)

Table 4. Crude and adjusted odds ratios of having no intention to vaccine their child 202 (N = 1,200)

**Discussion**

Add one paragraph on **Implication for policy and practice**
